# Creating a measure to operationalize engaged well-being at work

**DOI:** 10.1186/s12995-021-00297-0

**Published:** 2021-03-16

**Authors:** Catherin Bosle, Joachim E. Fischer, Raphael M. Herr

**Affiliations:** Mannheim Institute of Public Health, Social and Preventive Medicine, Mannheim Medical Faculty, Heidelberg University, Ludolf-Krehl-Strasse 7-11, 68167 Mannheim, Germany

**Keywords:** Mental well-being, Work engagement, Employees, Cluster analysis, Operationalization

## Abstract

**Background:**

Mental well-being and work engagement are both desirable, positive states of mind that help employees to better function in the workplace. While occupational researchers have argued in favor of considering both states concurrently, it is less clear how this might be translated to provide an instrument characterizing the workforce accordingly. The present study describes empirical efforts to operationalize a construct called engaged well-being.

**Methods:**

We used employee-level data (*n* = 13,538) from three waves of the German linked personnel panel (LPP; 2012–2017). Exploratory factor analysis and a combination of hierarchical and non-hierarchical cluster analyses linked with within-sum-of-squares statistics were used to identify distinct profiles describing mental well-being and work engagement concurrently. These profiles were then used as the basis to identify cut-offs to create replicable categories of engaged well-being. Using the longitudinal data from a subgroup providing data across more than one wave, we observed whether the newly constructed indicator changed over time.

**Results:**

The exploratory factor analysis suggested that both states were two distinct factors. Cluster analysis linked with within-sum-of-squares statistics suggested a four-cluster solution: engaged well-being (46.9%), disengaged well-being (27.5%), engaged strain (8.8%), and disengaged strain (16.8%). One cut-off for each state was identified to replicate the cluster solution. Across observation periods, we could observe changes in engaged well-being.

**Conclusions:**

Our measure of engaged well-being can be used to simultaneously characterize a workforce’s mental well-being and work engagement. Changes in this measure over time suggest its potential utility in organizational interventions. Future studies are needed to further explore both the antecedents, correlates, and potential effects of engaged well-being.

## Background

Occupational research has traditionally focused on reducing factors that cause stress and might lead to disease and infirmity, but an increasing emphasis on positive organizational behavior has shifted attention to individuals’ strengths and healthy functioning and the workplace conditions that facilitate them [1–3]. Mental well-being and work engagement are two desirable, positive states of mind that help individuals to better function in the workplace [4].

Both mental well-being and work engagement have individually received much attention. Based on a definition from the World Health Organization (WHO), an individual in a state of mental well-being “realizes his or her own potential, can cope with the normal stresses of life, can work productively and fruitfully, and is able to make a contribution to her or his community” [5]. Mental well-being is widely considered to have both hedonic (i.e., feeling good) and eudaimonic components (i.e., functioning well) [6, 7]. Employees with low mental well-being, for example, are more likely to be less productive and have more days of sick leave [8]. Moreover, in Germany mental disorders have been identified as one of the most common reasons for days of sick leave (16.6%) [9] and the most common health-related reason for early retirement (43%) [10]. Work engagement, on the other hand, is commonly defined as a work-related state of mind that is positive and fulfilling and not focused on a single object, event, or person [11]. It encompasses vigor (e.g., having high levels of energy, mental resilience, persistence), dedication (e.g., having a sense of significance, enthusiasm, inspiration, pride, challenge), and absorption (e.g., being fully concentrated and deeply engrossed, feeling time flies by, having difficulty to detach from work) [11]. Work engagement is associated with greater life satisfaction [12, 13], happiness [14], and better health outcomes [13, 15]. Other studies identify associations between work engagement and greater job satisfaction, better in-role and extra-role job performance, and lower absenteeism [13, 16–19]. Additionally, a meta-analysis has found that work engagement is related to better business outcomes such as customer satisfaction, productivity, profit, employee turnover, and accidents [20].

Occupational researchers have argued in favor of considering both states concurrently [1, 21, 22]. Even though the research listed above indicates that both states are relevant for both employees and employers, mental well-being is a state that focuses on life as a whole and is therefore thought to be particularly important for employees, while the work-related nature of work engagement makes it also especially relevant to employers [21]. Combining the employee perspective on well-being and health with the employer perspective on productivity has the potential to offer mutual benefit [1]. For example, conceptual work suggests that an indicator characterizing both mental well-being and work engagement might be a better predictor for the success of organizational interventions (e.g., coaching) than monetary outcomes such as return of investments [22]. Grant (2012) [22] reasons that when both states are considered simultaneously, they offer a more direct and holistic view of what most interventions intend to address – that is, improvements in the behaviour or state of employees that should in the long run lead to several organizational benefits. Other work suggests that a narrow focus on only one of these factors in organizational interventions as an intermediate outcome measure will limit the more distal organizational benefits of said interventions [4]. Indeed, their cross-sectional study indicates that mental well-being and work engagement are related states that simultaneously better explain variations in a common outcome of interest in organizational research: employee productivity [4]. In general, previous literature and established models such as the job-demands resources model suggest that in the workplace both health-related and motivational processes operate to influence not only employees but also organizational performance indicators [23–25].

Mental well-being and work engagement are positively associated with one another [26, 27], however, studies that address both states empirically are relatively sparse [28]. Even fewer studies have argued, how a concurrent consideration can be transformed to provide a single indicator that characterizes the workforce based on their mental well-being and their work engagement. Robertson and colleagues, for example, call for the addition of mental well-being to work engagement to obtain “full engagement” [4, 21, 29]. They argue that while many engagement scales already include items describing well-being, they are not comprehensive enough to sufficiently capture mental well-being and therefore require a separate indicator of mental well-being. Full engagement is therefore a combination of being engaged and experiencing high mental well-being [21]. However, while the authors establish that both states are moderately correlated, they do not demonstrate that they are distinct, even though both show independent associations with productivity [4]. Previous work such as the “well-being and engagement framework”, conceptualizes the presence of a conjoint construct but, to our knowledge, has resulted in no empirically defined measurements for the proposed categories [22]. This framework, for example, suggests that mental well-being and work engagement form a two-dimensional space in which the employees can be divided into four meaningful subgroups (flourishing, acquiescent, distressed but functional, distressed and disengaged). These subgroups characterize a workforce based on what combinations of high or low levels of mental health and work engagement employees are reporting [22, 30, 31]. To the best of our knowledge, the extent to which this two-dimensional room can be separated into four or, indeed, any finite number of categories has not yet been demonstrated using empirical data. A further knowledge gap is the absence of evidence that any operational measure for this construct demonstrates change over time: such evidence would be needed to justify its use as an intermediate outcome in interventions studies.

The present study describes empirical efforts to operationalize a construct characterizing a workforce’s mental well-being and work engagement, which we will refer to as “engaged well-being”. These efforts will address three aims. First, based on previous research [4], we quantify the extent to which mental well-being and work engagement are correlated and confirm that they are distinct states that can be used as two separate factors for further analysis. Second, assuming that these states are distinct, we test whether they can be divided into meaningful subgroups with distinct profiles. Although previous conceptual work hints at four subgroups, we will develop a categorization scheme that best fits data from a large database of employees. For this we use multiple statistical techniques and corresponding validation procedures to develop a robust taxonomy that identifies subgroups within a large sample that vary in potentially important ways with respect to the construct. Finally, we will use longitudinal data to test whether the newly constructed indicator can change over time.

## Methods

### Data

This study used the three waves of the Linked Personnel Panel (LPP; wave 1213, 1415, and 1617, DOI: 10.5164/IAB.LPP1617.de.en.v1), a longitudinal panel initiated by the German Federal Ministry of Labor and Social Affairs (BMAS) and administered at the Institute for Employment Research (IAB) [32–35]. The LPP links information on both the employer (e.g., human resources culture, management instruments) and employee (e.g., work characteristics, health status, sociodemographic characteristics). It is considered representative of private, moderate- to large-sized (> 50 employees) German companies in the manufacturing and service sectors [34]. The LPP was sampled from the Institute for Employment Research Establishment Panel, which is an annual representative survey of 16,000 German companies representing all industries and sizes nationwide [36, 37]. Companies from the business sectors of agriculture, forestry and fishery, as well as civil service and charity organizations or with less than 50 employees were excluded. The sample was stratified according to region, sector, and size [34, 36]. Data access to the LPP was provided via on-site use at the Research Data Centre (FDZ) of the German Federal Employment Agency (BA) at the IAB and subsequent remote data access.

Overall, the LPP contains data from 7508 employees and 1219 companies in the first wave (2012/2013), 7282 employees and 771 companies in the second wave (2014/2015), and 6779 employees and 846 companies in the third wave (2016/2017). Inclusion criteria for the present study were no missing values on the two indicators for mental well-being and work engagement, and working in a company with 50 or more employees (13,538 employees with 20,170 observations; 96.7% of all respondents). The analytic sample was limited to the first observation for each employee (*n* = 13,538), ensuring an equal weight for each individual both for the first and second aim. For the third aim, we needed longitudinal data and therefore we used the subgroup of individuals that were observed in at least two successive waves (*n* = 2891 between 2012 and 2014; *n* = 3528 between 2014 and 2016). Participants provided informed consent and the Ethics Committee of the Medical Faculty of the University of Heidelberg approved the use of the LPP for secondary data analysis (2018-514 N-MA).

### Measures

#### Mental well-being

Mental well-being was measured using the WHO-5 Well-Being Questionnaire (version 1998), a commonly used and validated instrument [38, 39]. This instrument consists of five items with responses rated on a 6-point Likert scale (0 ‘at no time’; 5 ‘all of the time’). Items assessed whether during the last 2 weeks employees felt ‘cheerful and in good spirits’, ‘calm and relaxed’, ‘active and vigorous’, ‘fresh and rested’, and whether their daily life was filled with things that interested them. In addition to using responses to individual items in our factor analysis (see below), we calculated an overall mental well-being index as the sum of the five items multiplied by four (range 0–100) for the remaining analyses. Higher values indicate a better assessment of one’s well-being with a value of ≥51.0 considered indicative of good mental well-being [39].

#### Work engagement

Work engagement was measured using a validated short-version of the nine-item Utrecht Work Engagement Scale (UWES-9) [11, 40, 41]. The UWES-9 measures responses on a Likert scale from 1 ‘never’ to 5 ‘daily’ to the following: ‘At my work, I feel bursting with energy’, ‘At my job, I feel strong and vigorous’, ‘When I get up in the morning, I feel like going to work’, ‘I am enthusiastic about my job’, ‘My job inspires me’, ‘I am proud of the work that I do’, ‘I am immersed in my work’, ‘I feel happy when I am working intensely’, and ‘I get carried away when I’m working’. In addition to using responses to individual items in our factor analysis (see below), a mean score (range 1–5) across all nine items was calculated, a higher score indicating greater work engagement. It must be noted, that the original UWES ranges on a scale from 0 to 6, however, other research indicates that the overall and all three sub-indices using the shortened scale show a similar internal consistency as the original work [34]. In line with the mental well-being scale, responses to individual items were used for the exploratory factor analysis and the overall score was used for all remaining analyses.

#### Descriptive sample characteristics

Individual characteristics used to describe the analytical sample were gender (male; female), age (in years), white-collar/blue-collar status (self-report), and full-time/part-time work.

### Analyses

Sample description was presented as the absolute (n) and relative (%) distribution of categorical variables, as well as mean values and standard deviations (S.D.) of all metric measures. We conducted our analyses using the statistical software package STATA, version 14 [42].

#### Aim 1: distinctiveness and correlation

Before operationalizing an indicator characterizing both mental well-being and work engagement, we first performed an exploratory factor analysis using a maximum likelihood estimation method with varimax rotation to assess whether items intended to reflect mental well-being and work engagement resulted in separate factors indicating two distinct states. We defined the number of factors using the Kaiser criterion (Eigenvalue of ≥1.0) and a factor loading of ≥ .3 was considered sufficient for assigning an item to a factor. We used Cronbach’s alpha to evaluate the internal reliability of the scales and Pearson’s correlation coefficients to assess the extent to which the overall scores of mental well-being and work engagement were correlated with each other.

#### Aim 2: defining meaningful subgroups

We used cluster analysis to assess the optimal number of categories and the respective cut-offs for engaged well-being in our dataset. A cluster analysis groups the analytical sample into several distinct clusters that include observations with similar profiles (i.e., similar combinations of levels of mental well-being and work engagement). An established clustering procedure was applied [43, 44]. The overall scores for mental well-being and work engagement were used as the two dimensions to define the profiles generated by cluster analysis. Because cluster analysis requires all indicators to have equal scales, both indicators were transformed to z-scores. As we made no assumptions on the number of categories a priori, we examined the possibility of multiple cluster solutions (k = 2, 3, …, 9). To guide identification of the cluster solution best fitting our data, we followed several steps. First, Ward’s hierarchical clustering was applied and these results were then used as the cluster centers for non-hierarchical k-means clustering. This two-step procedure is recommended because hierarchical models can lead to nonoptimal solutions [43]. That is, hierarchical models start with n clusters including one observation each. The two clusters with the smallest Euclidean distance are then combined in a stepwise procedure, thus reducing the number of clusters to n-1, n-2, …, n-(n-1) and increasing their size. However, once fused, individual observations or smaller clusters are not reassigned even if in later steps other cluster centers would present a better fit. The non-hierarchical procedure thus improves the clustering by (re-)assigning every observation to the cluster center that is most similar to the individual observation. Similarity was defined by the smallest Euclidean distance between individual values and the cluster centers provided by the Ward’s hierarchical clustering procedure. New cluster centers were then computed. Appendix A describes further analyses to test the agreement between the two clustering steps (Cohen’s *κ*) as well as an established double-cross validation procedure, that aimed to test the replicability (stability) of our k cluster solutions and to identify the best solution [44].

We applied a previously reported procedure to define the optimal k-means cluster solution (for more details see [45]). For each cluster solution, we calculated the within-sum-of-squares (WSS_k_), its natural logarithm [log (WSS_k_)], the eta-squared (η^2^_k_ = 1-WSS_k_/TSS) coefficient, and the proportional reduction of error coefficient [PRE_k_ = (WSS_k-1_-WSS_k_)/WSS_k-1_]. The η^2^-coefficient is an indicator for the proportional reduction of the WSS for a specific cluster solution compared to the total sum of squares (TSS), while the PRE-coefficient measures the proportional reduction of the WSS for a specific k-cluster solution compared with the next smaller cluster solution (k-1). These statistics indicate how the variance explained increases with the number of clusters. If the improvement of variance explained after a specific cluster solution levels off, larger cluster solutions should not be chosen [45].

To assure the reproducibility of our measurement for engaged well-being with the objective to address the third aim and for the sake of its utility in future studies, we used the results of the cluster analysis to define general cut-offs for the metric scores of the mental well-being and work engagement dimensions. These cut-offs are needed to divide the two-dimensional space created by the dimensions of mental well-being and work engagement into subgroups that reflect the results of the cluster analysis as closely as possible. Depending on the cluster solution (see below), we explored a series of different cut-offs. Our choices for cut-offs were primarily guided by A) the use of established cut-offs that can assign meaningful content to the clusters and B) the use of deciles to identify cut-offs with an approach that balances the precision of the cut-offs and the complexity and extent of the analysis. We create several indicators comprising the categories (i.e., subgroups) based on these cut-offs and test them against the cluster solution. Cohen’s *κ* as well as the proportion of agreement between each newly generated indicator and the cluster solution were used to identify the indicator with the highest agreement in comparison with the cluster solution. To compare the categories of the indicator of engaged well-being with the results of the cluster solution, we provided a description of their profiles (means of mental well-being and work engagement), cross-tabulation and chi^2^-testing, as well as Cohen’s *κ*. This indicator was chosen for further subgroup analysis (see below).

#### Aim 3: changes over time

Using the newly defined indicator for engaged well-being and its cut-offs, we assigned the categories of engaged well-being for the 2nd and 3rd observation of the subgroup of employees that participated in at least two successive waves. Using this longitudinal information (wide format), we described whether and how employees changed categories across time. Changes are presented as the migration between categories from one observation point to the next (%).

## Results

### Description of analytical sample

Table 1 provides descriptive statistics of the analytical sample. Mental well-being was on average rated as good (62.25, ±20.83) and the average reported work engagement is located in the upper third of the total scale (3.73, ±0.81). The sample had a mean age of 45.96 years (±10.87), was primarily male (71.28%), consisted predominantly of white-collar workers (62.53%) and of employees working in full-time positions (86.74%).

**Table 1:** Descriptive statistics of the analytical sample (13,538 employees)

	mean / %	S.D. / n
mental well-being (range 0–100)	62.25	20.83
work engagement (range 1–5)	3.73	0.81
age (years)	45.96	10.87
male	71.28	9650
female	28.72	3888
white-collar	62.53	8461
blue-collar	37.47	5071
full-time	86.74	11,731
part-time	13.26	1793

### Aim 1: distinctiveness and correlation

The EFA using all items for mental well-being and work engagement provided a two-factor solution showing that they were distinguishable constructs with all items of the WHO-5 scale having a higher loading (≥ .3) on one factor and all items of the UWES-9 having a higher loading (≥ .3) on the other factor. No cross-loadings were present and therefore each item can be attributed to one single factor. Cronbach’s alpha for mental well-being and work engagement were .851 and .909, respectively, indicating very good internal consistencies. Both constructs correlated moderately (Pearson’s r = .398; *P* < 0.001).

### Aim 2: defining meaningful subgroups

Table 2 presents the results of the within-sum-of-squares statistics. The larger the number of clusters, the smaller the WSS and therefore the greater the variance explained. The decreases in WSS are, however, much weaker after the four-cluster solution. Additionally, the PRE-coefficients indicate that the percentage improvement in WSS indicators is much lower after the four-cluster solution (15% improvement between the four- and five-cluster solution compared to 25% improvement between the three- and four-cluster solution) and levels off afterwards. A similar drop in improvement can be found after the 2-cluster solution, however, here the reduction of the WSS with an η^2^-coefficient of 46% is comparatively small. The double-cross validation presented in Appendix A indicates that the 2- and 4-cluster solutions were most replicable (stable).

**Table 2:** Within-sum-of-squares statistics by number of clusters (*n* = 13,538)

cluster solution (k)	WSS_**k**_	log (WSS_**k**_)	η^**2**^_**k**_	PRE_**k**_
1	27,680.66	10.23	.00	.
2	14,959.98	9.61	.46	.46
3	11,250.22	9.33	.59	.25
4	8394.58	9.04	.70	.25
5	7102.47	8.87	.74	.15
6	6071.34	8.71	.78	.15
7	5091.35	8.54	.82	.16
8	4560.92	8.43	.84	.10
9	4168.98	8.34	.85	.09

Note: The statistics are calculated as proposed by Makles (2012) [45]; *WSS*_*k*_ within-sum-of-squares; *log (WSS*_*k*_*)* the natural logarithm of WSS_k_; *η*^*2*^_*k*_ 1-WSS_k_/WSS_1_ [eta-squared]; *PRE*_*k*_ (WSS_k-1_-WSS_k_)/WSS_k-1_ [the proportional reduction of error]

**Fig. 1:**
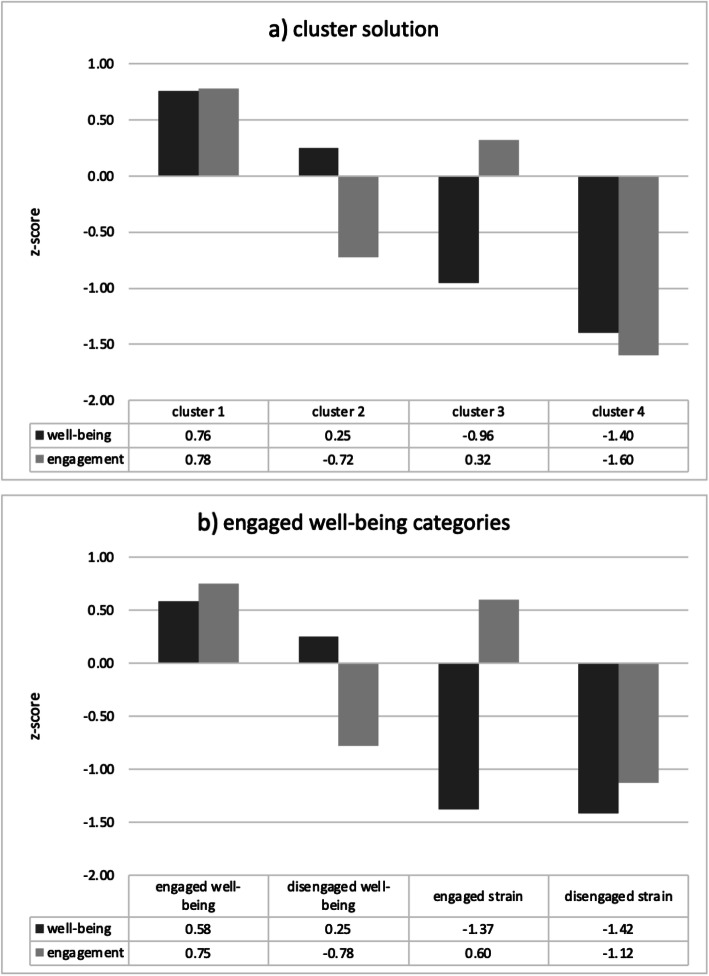
Profiles of **a**) the cluster solutions and **b**) the engaged well-being categories (values transformed to z-scores, n = 13,538).

We therefore defined cut-offs for categories comprising engaged well-being using the four-cluster solution. Figure 1a presents the description of the cluster profiles using values of mental well-being and work engagement that were transformed to z-scores. The first cluster exhibits on average both higher mental well-being and work engagement. The last cluster, on the other hand, has both lower well-being and work engagement. The other clusters exhibit either high mental well-being and low work engagement or low mental well-being but high work engagement. Each cluster thus occupies one corner of the two-dimensional space that mental well-being and work engagement form. Based on this distribution, we identified one cut-off for each dimension to create categories that closely matched the cluster solution. Because mental well-being measured by the WHO-5 already has an established cut-off indicating good mental well-being (≥ 51.0), we test this cut-off for the first dimension. As for the second dimension – work engagement – no cut-offs exist, so we chose to use values corresponding to each of the nine deciles. We then generated nine different indicators of engaged well-being, each including four categories based on the established cut-off for mental well-being and one of the nine cut-offs for work engagement. Appendix B Table 1 presents the agreement between several indicators of engaged well-being, based on different cut-offs, and the four-cluster solution. With an overlap of 80.74% and a Cohen’s *κ* of .728 the indicator using the 4th decile of the work engagement distribution and the established cut-off for mental well-being provided numerical values that most closely corresponded to the solution identified by cluster analysis.

Figure 1b provides a description of the indicator of engaged well-being that had the highest agreement with the cluster solution. We labelled the first category ‘engaged well-being’ as it exhibits on average both higher mental well-being and work engagement. The last category, with both lower well-being and work engagement was labelled ‘disengaged strain’. The remaining clusters were labelled ‘disengaged well-being’ (high mental well-being and low work engagement) and ‘engaged strain’ (low mental well-being but high work engagement).

Further comparison of the distribution of both engaged well-being and the results of the cluster analysis also revealed a good fit (Table 3). Clusters 1, 2, and 4 are largely assigned to a single category of engaged well-being: engaged well-being (96.88%), disengaged well-being (92.90%), disengaged strain (86.78%), respectively. Cluster 3, best resembles the category engaged strain (40.18%). The comparison shows a highly significant association (*P* < 0.001) between the cluster solution and the engaged well-being categories. Table 4 illustrates the categories of engaged well-being.

**Table 3:** Agreement between the four-cluster solution and the indicator for engaged well-being (% of employees, n = 13,538)

		results of cluster analysis			
		cluster 1	cluster 2	cluster 3	cluster 4
**engaged well-being**	engaged well-being	***96.88***	4.04	30.45	0.00
	disengaged well-being	3.12	***92.90***	6.57	13.22
	engaged strain	0.00	0.00	***40.18***	0.00
	disengaged strain	0.00	3.06	22.80	***86.78***
	total	100	100	100	100

**Table 4:** Description of the categories of engaged well-being

	***mental well-being***			***work engagement***		
	defined as		cut-off at	defined as		cut-off at
***engaged well-being***	+	good to very good	a value of ≥51.0	+	good to very good	top 60% (a value of ≥3.7)
***disengaged well-being***	+	good to very good	a value of ≥51.0	–	reduced	lowest 40% (a value of ≥3.7)
***engaged strain***	–	reduced	a value of < 51.0	+	good to very good	top 60% (a value of < 3.7)
***disengaged strain***	–	reduced	a value of < 51.0	–	reduced	lowest 40% (a value of < 3.7)

### Aim 3: changes over time

**Fig. 2:**
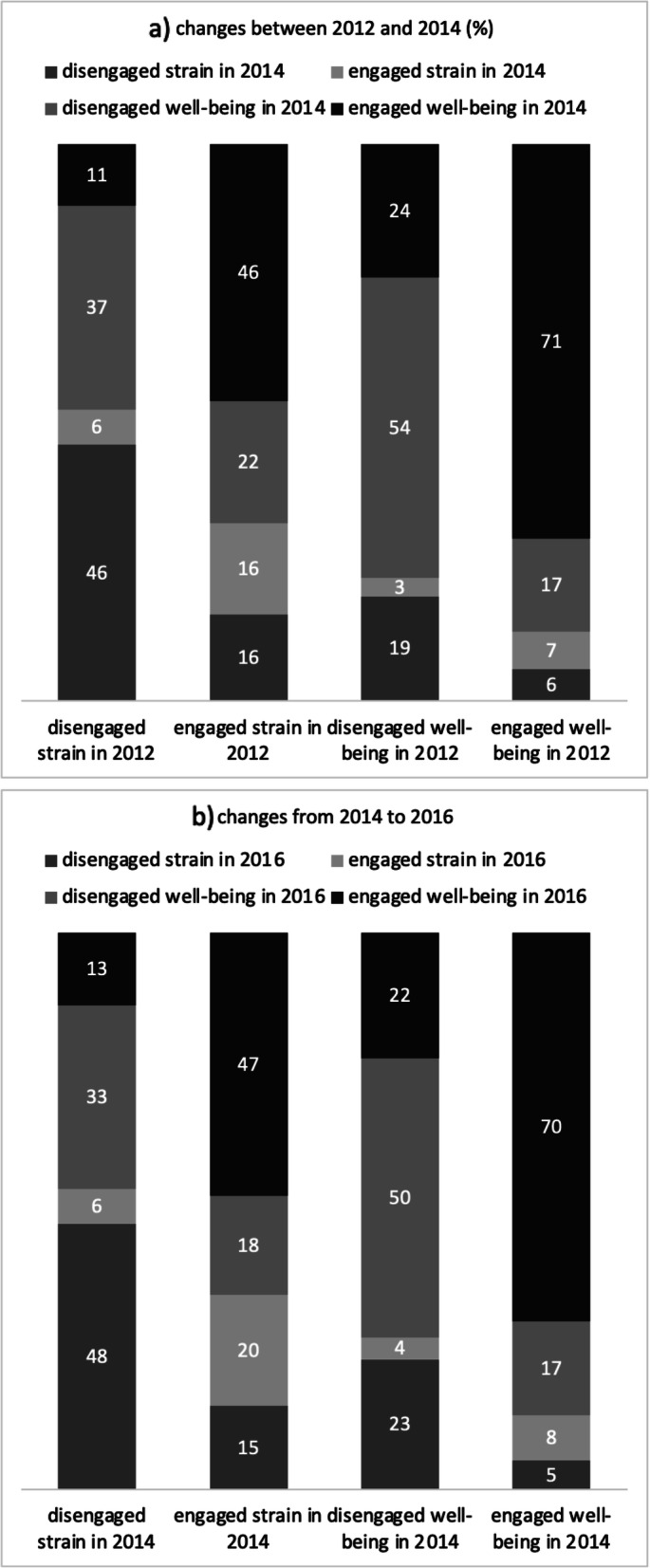
Individual changes in engaged well-being between the observations 2012/2014 and 2014/2016 (in %). Note: employees with at least one missing observation in 1 year were excluded

Figure 2a and b present individual changes in engaged well-being over time (2012 to 2014; 2014 to 2016) when applying the cut-offs to the 2nd and 3rd observation of the longitudinal data. Both tables show similar changes. Most employees in the category engaged well-being also reported this category in the next year (70.82% / 69.88%). Employees that reported to be disengaged strained in the first year, mostly reported the same in the second year (45.93% / 47.74%) or reported a change into the category “disengaged well-being (36.70% / 33.09%). On the other hand, employees that reported to be engaged but strained in the first year, most often reported engaged well-being in the second year (46.22% / 47.30%). About half of the employees that belong to the category disengaged well-being in one observation, also report this category in the next observation (54.07% / 50.19%). However, most of the employees that changed between two observation, changed either to disengaged strain (18.77% / 23.34%) or engaged well-being (23.88% / 22.49%).

## Discussion

Previous literature has proposed a simultaneous consideration of both mental well-being and work engagement and various studies indicate that both states separately are associated with desirable outcomes for employees and employers. Using a large sample of employees, the present study added to previous research and considerations in three ways. First, additional support was provided that mental well-being and work engagement are moderately correlated. We have added to these correlations by providing evidence that both states are distinct. Second, it was tested whether these states can be divided into distinct subgroups by identifying profiles varying with respect to their average mental well-being and work engagement. The resulting subgroups can be described as 1) high mental well-being and high work engagement (engaged well-being), 2) high mental well-being and low work engagement (disengaged well-being), 3) low mental well-being and high work engagement (engaged distress), and 4) low mental well-being and low work engagement (disengaged distress). Replicability of the subgroups (or categories) was ensured by identifying and testing empirical cut-offs. The final construct is referred to as engaged well-being. Finally, we used longitudinal data to show that engaged well-being can change over time, indicating its potential use for intervention.

Our analyses mirror several conceptual considerations and previous empirical observations. First, in line with previous literature, we found that both mental well-being and work engagement are moderately and positively correlated [4, 46]. The positive correlation between both constructs was to be expected as a) the work engagement scale includes items that describe well-being at work, such as “I feel happy when I’m working” and that b) previous studies have found significant positive associations both cross-sectionally and longitudinally [26, 27]. Additionally, we have shown that both states are distinct, which supports a previous analysis showing that mental well-being and work engagement have distinct associations with productivity [4].

Our findings additionally correspond with ideas formulated in the ‘engagement and well-being framework’, arguing that the dimensions mental well-being and work engagement can be comprised into four different categories occupying every corner of this two-dimensional space [22, 31]. However, we did not predefine the number of categories for engaged well-being and instead chose established, data-driven approaches testing multiple cluster solutions. Still, the best solution provided in our analysis was the four-cluster solution. In line with the ‘engagement and well-being framework’, the resulting clusters or categories combine either higher or lower levels of mental well-being and work engagement. A notable distinction to the framework is that it defines the mental well-being dimension as a combination of mental well-being and mental illness [22]. Our study uses the WHO-5, a generic scale with only positively formulated items that reflect mental well-being and not mental illness [39]. The established cut-off is often used for screening in clinical depression trials, however, the WHO-5 itself has no diagnostic specificity [39]. In line with ideas from the positive psychology approach, which argues that the good is more than the absence of the bad [3], and based on previous literature that has shown that mental health and mental illness [47], as well as positive and negative affect [48] cannot be measured on a single dimension, we decided against a combination of both positive and negative health-related states in the mental-well-being dimension. Measuring mental illness separately or possibly as another dimension in engaged well-being could be valuable, especially because positive organizational psychology studies indicate that positive and negative phenomena explain unique variance of organizational outcomes [1]. We do believe that engaged well-being could be an instrument used within the mental well-being and engagement framework.

Engaged well-being combines interests of employees and employers [1]. While the motivational processes associated with work engagement are often the main focus for employers, taking employee health into account is not only part of the corporate social responsibility but also of business interests, as healthy employees are more productive and the image of an organization that takes care of its employees is likely to increase [49]. One study proposes that, due to the positive association with productivity, organizations need to apply more holistic and multipronged approaches to improve work engagement and physical health by creating motivational work environments and providing health and wellness programs [17]. Positive psychology interventions seem to be promising for enhancing both employee well-being and performance [50]. While the present study is the first step in corroborating a simultaneous consideration of mental well-being and work engagement in the form of engaged well-being, we are aware that more work is needed to strengthen the construct and to establish it in organizational settings, especially in interventions. In the following section, we therefore discuss the application and further testing of engaged well-being.

### Future research and application in organizational settings

Our operationalization of engaged well-being is easily replicable and can change over time, giving it potential to be applicable in workplace interventions. The approach we have described in measuring engaged well-being could be used in organizations to observe the distribution of employees across the different categories as well as the changes of this distribution over time. However, we still need to test, what antecedents lead to these changes. As proposed in the job-demands resources model, there are two pathways through which job characteristics influence employees – the health-impairment and the motivational pathway [23]. The model assumes, that while job resources are thought to be predominantly positively associated with work engagement through the motivational process, job demands are mainly negatively associated with mental well-being through the health-impairment process. Several empirical studies have found support for the assumptions of this model (for reviews see [23, 25, 51]). Based on these pathways, we assume different needs for changes between different engaged well-being categories. For example, employees in the category engaged strain might be more likely to change into the category engaged well-being if job demands (e.g., physical demands, time pressure, mobbing) are reduced, as this should increase their mental well-being (health-impairment process). Employees in the category disengaged well-being, on the other hand, might be more likely to change into the category engaged well-being if job resources (e.g., supportive leadership, organizational justice, decision-making autonomy) are increased, as this should increase their work engagement (motivational process). These assumptions also imply that not all employees within an organization would need the same type of support, depending on their engaged well-being, therefore implying multi-component interventions. However, while the direct associations described above have been shown in previous research, multiple studies using the job-demands resources model have also shown interaction effects between demands and resources [23, 25, 49]. It is assumed that job resources do not only affect work engagement through the motivational process, but that employees with increased job resources are additionally better able to cope with the strain caused by job demands, therefore reducing their negative impact on health [23, 25]. A more distinctive analysis of such interactions regarding engaged well-being is important to better understand the antecedents and processes that influence this new construct.

Workplace interventions can be used to test whether and how changes in job demands or resources can influence engaged well-being. By improving work conditions (e.g., increasing supervisory support or decreasing time-pressure), employers should be able to observe a shift away from disengaged strain towards engaged well-being. It is, however, important to note, that while the overall conceptual thoughts of the ‘engagement and well-being framework’ might be translated to both the employee and the organizational level [22, 30], the use of engaged well-being within workplace interventions should be limited to observing changes within the overall workforce of an organization rather than within an employee, as the engaged well-being categories are rather broad and therefore not able to provide detailed information on changes within individuals.

Within such workplace interventions, a deeper understanding of the two categories of engaged strain and disengaged well-being needs to be developed. Why do people report being engaged while they are strained? One conclusion might be that these employees could be addicted to their work and thus risking their own mental well-being. However, work engagement has been defined as a positive state of mind and studies indicate that work engagement and workaholism are two different constructs [13, 52]. Additionally, our results indicate that only every fifth employee that had been engaged strained in one observation reported the same in the next, and every second reported an improved change to engaged well-being, indicating that it might not be the higher levels of work engagement that result in strain. In contrast, the category disengaged well-being was more stable. Are these employees that do not care for their work and search for validation outside of the work environment? How can changes to engaged well-being still be encouraged (e.g., through better supervision)?

Additionally, a better understanding of the consequences of engaged well-being makes the indicator more attractive for use in praxis. As argued by Grant (2012) [22], indicators that capture employee level engagement and well-being might be better indicators of organizational success than monetary business outcomes. In the short run monetary outcomes could be quickly improved by worsening working conditions (e.g., high pressure work environments). In the long run, engaged well-being should lead to more organizational success, as employees should have better resources to reach organizational goals and are less likely to ‘burn out’. Previous studies have found positive and distinct associations of mental well-being and engagement with productivity cross-sectionally [4] or longitudinally using a physical instead of a mental health indicator [17]. Future studies should test this assumption by analysing the long-term effects of engaged well-being on productivity and other indicators of organizational success.

We furthermore need to discuss the interpretation of the categories of engaged well-being in relation to one another. It can be assumed that it is the least desirable to have many employees in the category disengaged strain that has on average the lowest ratings of mental well-being or work engagement, while engaged well-being should be the most desirable category. Whether the category disengaged well-being or engaged strain is “preferable” cannot be clearly defined. An ordinal or metric interpretation is therefore not possible. However, because we assume that changes in different antecedents (i.e., work characteristics) have different consequences depending on the category of engaged well-being employees find themselves in, this distinction between disengaged well-being and engaged strain is necessary for employers to make informed decisions.

### Strengths and limitations

A strength of the present study is its use of established and validated indicators of mental well-being and work engagement [39, 41]. However, the generalization of our work using the UWES-9 for work engagement is somewhat limited due to the data including a shortened scale compared to that of the original work. Future work needs to test, whether similar findings can be found using the original scaling. The use of established clustering procedures that are accompanied by several sensitivity analyses (e.g., double-cross validation, within-sum-of-squares statistics) is another strength. Because our complete case analysis was based on only two indices, we were able to include 96.7% of all respondents in our cross-sectional analyses and we can assume that the selection bias due to missing data is rather small [53]. The bias might be larger for the longitudinal analysis, as we face sample attrition (e.g., due to a healthy worker bias [53]). Additionally, while on the employer level the LPP is representative for private, moderate- to large-sized German companies in the manufacturing and service sectors and employees from a wide variety of sectors and business sizes are included, the employee sample itself is primarily male, older, and working full-time, and results should therefore be interpreted carefully as they might not be representative for certain working populations. Future studies should therefore test our cut-offs using study populations with different sociodemographic characteristics. A bias due to common method variance cannot be excluded, as all items were measured subjectively and based on self-reports [54]. Therefore, we propose to further test engaged well-being against objective indicators, such as biomarkers that are associated with stress or objective indicators for productivity.

## Conclusion

Our measure of engaged well-being can be used to simultaneously characterize a workforce’s mental well-being and work engagement. Change in this measure over time suggests its potential utility in organizational interventions. Future studies are needed to further explore both the antecedents, correlates and potential effects of engaged well-being.

## Data Availability

The data that support the findings of this study are available from the Research Data Centre (FDZ) of the German Federal Employment Agency (BA) at the Institute for Employment Research (IAB) but restrictions apply to the availability of these data, which were used under license for the current study, and so are not publicly available. Data access can be requested from the Research Data Centre (FDZ) of the German Federal Employment Agency (BA) at the Institute for Employment Research (IAB).
